# Estrogen Activity in Plastic Products: Yang et al. Respond

**DOI:** 10.1289/ehp.1103894R

**Published:** 2011-09-01

**Authors:** Chun Z. Yang, George D. Bittner, Stuart I. Yaniger, Daniel J. Klein, V. Craig Jordan

**Affiliations:** CertiChem Inc., Austin, Texas; PlastiPure Inc., Austin, Texas; Lombardi Comprehensive Cancer Center, Georgetown University Medical Center, Washington, DC

In their letter, Kelce and Borgert raise points related to our methods, as well as the objective of our paper (Yang et al. 2011) and its significance.

Regarding our methods, our solvent extraction procedures were less stringent than U.S. Food and Drug Administration (FDA)-recommended methods for determining migration from plastic food packaging [37°C for 72 hr in our study (Yang et al. 2011) compared with 40°C for 240 hr for comparable FDA procedures (FDA 2002, 2007)]. Consequently, if we had used FDA-recommended procedures, we would expect to detect a higher frequency of chemicals with estrogenic activity (EA) leaching from plastic containers. At present, the FDA has no established standards regarding extraction of chemicals having endocrine-disrupting effects, including estrogenic activity (EA). In addition, Wagner and Oehlmann (2010) confirmed our data for polyethylene terephthalate (PET) plastics, moot other points made by Kelce and Borgert regarding our extraction procedures, and discussed the significance of such data in terms very similar to ours.

Kelce and Borgert question our method of using ultraviolet (UV) light as a stressor. In our study (Yang et al. 2011), UV exposures were only to one side of the plastic. The FDA has no established standards regarding exposure of food packaging to UV light. Because food packaging and containers are often exposed to various sources of UV light (e.g., sunlight, sterilization, high intensity UV curing of package decoration), we believe that a realistic evaluation of packaging hazards should include UV exposure, even absent specific FDA requirements.

Our resin data (resins P1. P2, P3, P4, P19, and P18) cited by Kelce and Borgert came from at least three replications of stressing, extraction, and EA assays. As described in our “Methods” and “Supplemental Material,” the assay variance was very small: SEs were typically smaller than the diameter of the data points of the graphed means. The whole series of 49 assays was repeated only once, but no extract exhibited EA; more recent extracts of the same plastics confirm our original results.

Kelce and Borgert noted that colorants are “embedded” in plastics. However, “bound” colorants in plastic compounds can and do readily leach from plastics. They are additives, which—like most additives—are only rarely chemically bound to polymers. Hence, concerns about all additives are warranted because any can leach from a plastic product.

Regarding broader issues, the objective of our paper was to quantify the prevalence of xenoestrogen release from commonly used plastic products. These data are significant in part to help assess the risk of such products to human health and environmental contamination. Kelce and Borgert cite Charles et al. (2007), who examined some interactions between a small set of phytoestrogens and xenoestrogens. The limited negative results of that study have been contradicted by dozens of other studies (e.g., Patisaul and Jefferson 2010). However, our objective was not to establish definitive links between public health issues, environmental pollution, and exposure to xenoestrogens. This relationship is an active research area, and it will take many years to obtain definitive answers.

Kelce and Borgert’s concerns about the paucity of epidemiological data correlating EA exposure via use of plastics with adverse human health effects is analogous to the long-standing controversy for tobacco, which is now highly regulated, largely because increasing numbers of epidemiological studies  correlated smoking with heart disease and lung cancer. For decades, it was common to hear tobacco industry spokespersons argue that “[epidemiological] correlation does not mean causation” and demand that molecular, cellular, and/or systemic mechanisms be extensively demonstrated before any action, regulatory or otherwise, be taken. One rarely hears spokespersons for the chemical and plastics industry make this argument for release of chemicals having EA from plastics, because the mechanisms by which tobacco has its effects are still much less well known compared to mechanisms by which chemicals having EA produce adverse health and environmental effects. Instead, we hear, “Where are the epidemiological correlations?” Those correlations are fewer (but not nonexistent) than for tobacco at this relatively young stage of the field, but the number of such publications is rapidly increasing. In the meantime, our study and hundreds to thousands of other *in vitro* studies demonstrate that chemicals having EA have easily measurable effects on all sorts of human cells (including MCF-7 cells). Most scientists in this field believe that such results suggest adverse health effects in humans and that, as such data continue to be gathered, these correlations will become as compelling as did those for the impact of tobacco smoking on public health.

Legislators, consumers, manufacturers, and scientists must judge current industry practices in this area based on available data. Reasonable people can differ. The American Chemistry Council takes the position that until definitive studies consistently show health and environmental hazards from chemicals with EA leaching from plastic products, no industry action need be taken. We disagree. Plastic items are essential consumer products, but we argue that they need to be made safer. Our most recent data show that there is very little extra expense to produce safer plastics that do not leach chemicals having EA; that is, it costs very little at this time to avoid a potential health risk.
